# Ectomycorrhizal Symbiosis as a Bio-Enhancement Strategy for Transplantation of Somatic Embryo-Derived *Pinus elliottii*

**DOI:** 10.3390/plants15111701

**Published:** 2026-05-30

**Authors:** Zhen-Xing Tian, Xin Ke, Xin-Yi Ji, Xi-Yuan Chen, Li-Hua Zhu

**Affiliations:** 1 College of Forestry and Grassland, Nanjing Forestry University, Nanjing 210037, China; tianzx@njfu.edu.cn (Z.-X.T.); 15251856007@163.com (X.K.); 3230100079@njfu.edu.cn (X.-Y.J.); xiyuanchen@njfu.edu.cn (X.-Y.C.); 2 Co-Innovation Center for Sustainable Forestry in Southern China, Nanjing Forestry University, Nanjing 210037, China

**Keywords:** somatic embryogenesis, *Pinus elliottii*, acclimatization, bio-enhancement, ectomycorrhiza, *Pisolithus orientalis*, plantlet quality

## Abstract

Somatic embryo-derived plantlets of pines often fail to survive acclimatization, which limits commercial micropropagation. Conventional hardening methods do not correct the physiological weaknesses of in vitro plantlets, especially the lack of beneficial microbes. Here we developed a practical protocol for resistant *Pinus elliottii*. First, we used an optimized maturation protocol (three sequential ABA pre-treatments) and glucose for germination. Substrate screening showed that a peat:vermiculite:perlite mixture (3:1:1) gave the highest survival (98.9%). Then, before transplantation, we introduced a key bio-enhancement step: in vitro inoculation with the ectomycorrhizal fungus *Pisolithus orientalis* cfcc7668. This treatment achieved a mycorrhization rate of 97.7% and transformed root morphology from thin, sparsely branched roots to a coralloid, dichotomously branched system with a well-developed Hartig net. As a result, mycorrhizal plantlets had 100% transplant survival at 30 days and remained above 94% over 360 days, whereas non-inoculated controls dropped to 95.6% at 30 days and further declined to about 73% after three months. Pre-establishing ectomycorrhizal symbiosis effectively restores a key root function missing in in vitro plantlets. Our integrated procedure provides a practical method for clonal propagation of conifers.

## 1. Introduction

The clonal propagation of superior conifer genotypes is a cornerstone of modern forest tree improvement programs, enabling the capture and mass deployment of valuable genetic gains [1]. Among the available biotechnologies, somatic embryogenesis (SE) stands out as a powerful tool for rapid clonal propagation, offering advantages such as fast multiplication and large-scale production of genetically uniform, disease-resistant plantlets [2]. Beyond these intrinsic advantages, SE is increasingly recognized as a pivotal technology for implementing advanced breeding and deployment strategies. As the global forestry sector transitions towards a bioeconomy with increased demands for biomass and climate change mitigation, the vegetative propagation of elite genotypes via SE provides an effective delivery mechanism for genetically superior materials, thereby enhancing both breeding accuracy and the scale of improved reforestation efforts [3].

The journey of conifer SE began with pioneering reports in spruce and larch in the mid-1980s [4,5]. Since then, remarkable progress has been made, particularly within the economically crucial genus *Pinus*. SE technology has now been successfully applied to a significant number of pine species. A growing body of literature indicates that successful SE has been reported in at least 36 *Pinus* species, including major timber trees such as loblolly pine (*P. taeda*), maritime pine (*P. pinaster*), masson pine (*P. massoniana*), radiata pine (*P. radiata*), and slash pine (*P. elliottii*) [6,7,8,9,10].

However, this impressive progress in laboratory regeneration masks a series of persistent bottlenecks that severely limit operational deployment. A critical challenge is the maintenance of embryogenic potential, which is often gradually lost with repeated subculturing, thereby impacting the reliable production of somatic embryos [11]. More critically, even when embryogenic tissues are successfully initiated, their subsequent development is frequently hampered. As highlighted in a recent review focusing on *Pinus* spp., while protocols for initiating embryogenic mass (EM) are now relatively well established, the successful maturation of EM into cotyledonary normal somatic embryos remains inconsistent [12]. This bottleneck manifests as low yields of high-quality mature embryos, asynchronous development, and abnormal morphology, and poor root development, which collectively lead to poor plantlet conversion rates [12,13]. Consequently, the ultimate and most visible failure point remains the frequent inability of somatic plantlets to survive the transition to ex vitro conditions—a well-documented phenomenon known as “acclimatization shock” [14,15].

The vulnerability of these plantlets is a direct consequence of physiological and anatomical frailties instilled by the in vitro environment [16]. They often exhibit poor photosynthetic competence, malfunctioning stomata, and underdeveloped cuticular waxes, rendering them highly susceptible to desiccation [17,18]. Perhaps most critically, their root systems are simplistic, poorly lignified, and functionally inadequate for efficient water and nutrient uptake from a non-sterile soil substrate [19,20]. Compounding these inherent weaknesses, the axenic nature of tissue culture means these plantlets are launched into the world devoid of beneficial microbial symbionts. Notably, they lack associations with ectomycorrhizal (ECM) fungi, which are crucial partners for the growth, nutrient acquisition, and overall stress resilience of pines in natural settings [21,22]. Consequently, upon transplantation, the somatic plantlets encounter severe water stress, low energy reserves, and increased susceptibility to soil pathogens [23].

Conventional strategies to improve acclimatization have primarily focused on environmental hardening and substrate optimization [24,25]. While necessary, these approaches often provide only marginal improvements, creating a “softer landing” but failing to address the fundamental functional deficiencies within the plantlet itself [26]. New approaches are needed that not only protect the plantlet but also improve its basic functions. This has led to the exploration of pre-establishing symbiotic relationships before transplanting. The integration of ECM fungi into micropropagation protocols, a process termed in vitro mycorrhization, has thus emerged as a bio-hardening approach [27,28]. Inoculation with compatible ECM fungi, such as *Pisolithus* spp., has been demonstrated to promote soil water and nutrient absorption, enhance tolerance to abiotic stresses, and increase resistance to pests and diseases [29,30,31]. This approach effectively prepares the plantlet for the rigors of the ex vitro world by providing it with a pre-assembled, functional support system [32,33].

Therefore, this study was designed to systematically address the multifaceted problem of acclimatization in slash pine. While robust protocols for somatic embryogenesis in *P. elliottii* have been established [10], the acclimatization of somatic plantlets remains a paramount bottleneck. We hypothesized that overcoming this barrier requires an integrated protocol that builds upon the production of high-quality somatic plantlets and biologically enhances their resilience through early ectomycorrhizal symbiosis. The core innovation of this study is the pre-transplant in vitro mycorrhization, which we introduce as a key bio-enhancement step to overcome the acclimatization bottleneck. We investigated the individual and combined effects of key factors spanning the entire process: from leveraging established maturation methods [10] and selecting for initial plantlet vigor, to fine-tuning the transplant substrate composition, and most critically, forging a symbiotic partnership with the ECM fungus *P. orientalis* cfcc7668. Our overarching goal was to develop a cohesive and reliable system that transforms the acclimatization stage from a stochastic bottleneck into a predictable, high-throughput process.

## 2. Results

### 2.1. Production and Selection of Vigorous Somatic Embryo-Derived Plantlets

**Figure 1:**
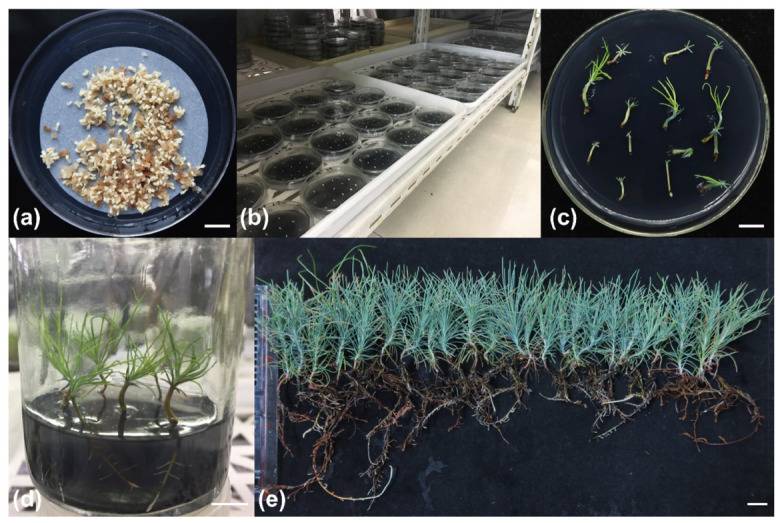
Production and selection of *Pinus elliottii* somatic embryo-derived plantlets. (**a**) Cotyledonary somatic embryos with normal morphology obtained. (**b**) Somatic embryos placed in Petri dishes containing germination medium. (**c**) Germinated somatic embryos with elongated hypocotyls and opened cotyledons (approximately half have developed radicles). (**d**) Intact plantlets with well-formed roots developed on rooting medium in a glass culture vessel. (**e**) Selected plantlets measuring 2.5–4.0 cm in height for transplantation experiments. Bar = 1.0 cm.

After 75 days of maturation culture, somatic embryos developed normally and produced cotyledonary embryos with normal morphology, yielding 352 ± 25 cotyledonary embryos per Petri dish (mean ± SD, *n* = 4) (Figure 1a). The mature cotyledonary embryos were then transferred to Petri dishes containing germination medium supplemented with 20 g/L glucose (Figure 1b). After one month on germination medium, approximately 90% of the somatic embryos showed elongated hypocotyls and opened cotyledons. Of these, only about 50% developed radicles (Figure 1c). The resulting seedlings were transferred to rooting medium and developed into intact plantlets with well-formed roots under in vitro conditions (Figure 1d).

To ensure uniform starting material for subsequent experiments, regenerated plantlets were further evaluated based on height and overall vigor. Plantlets measuring shorter than 2.5 cm generally exhibited reduced vigor and were excluded. Only plantlets within the 2.5~4.0 cm height range were therefore advanced for transplantation and acclimatization experiments (Figure 1e). From three independent maturation runs, a total of 360 plantlets met this height criterion and were used for subsequent experiments.

### 2.2. Effects of Substrate Composition on Transplant Survival

**Figure 2:**
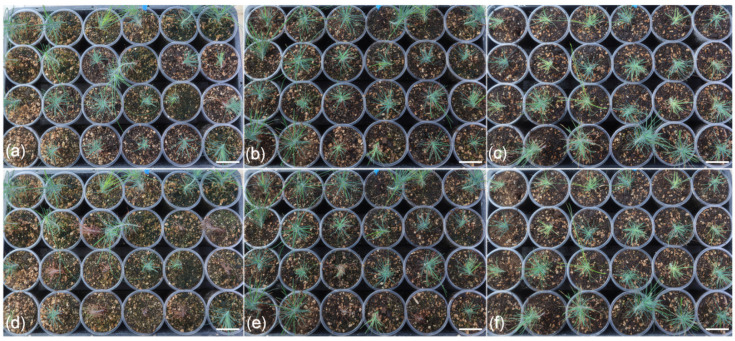
Representative growth and survival of regenerated *Pinus elliottii* plantlets grown in substrates with different compositions. (**a**–**c**) Plantlets transplanted into peat:vermiculite:perlite substrates at ratios of 1:1:1, 2:1:1, and 3:1:1, respectively. (**d**–**f**) Survival of plantlets grown in the 1:1:1, 2:1:1, and 3:1:1 substrates 30 days after transplantation, respectively. Bar = 5.0 cm.

Substrate composition significantly influenced the survival of regenerated plantlets following transplantation (Table 1, Figure 2). Survival was lowest in the peat:vermiculite:perlite substrate at a 1:1:1 ratio (66.7%, 60/90). Increasing the proportion of peat markedly improved transplant success, with survival reaching 93.3% (84/90) in the 2:1:1 substrate and 98.9% (89/90) in the 3:1:1 substrate 30 days after transplantation. Statistical analysis indicated that survival in both the 2:1:1 and 3:1:1 substrates were significantly higher than that observed in the 1:1:1 substrate, whereas no significant difference was detected between the 2:1:1 and 3:1:1 treatments (Table 1). These results indicate that a substrate providing an appropriate balance between moisture retention and aeration is critical for mitigating transplant stress during the early ex vitro phase.

### 2.3. Mycorrhizal Establishment and Transplant Survival

**Figure 3:**
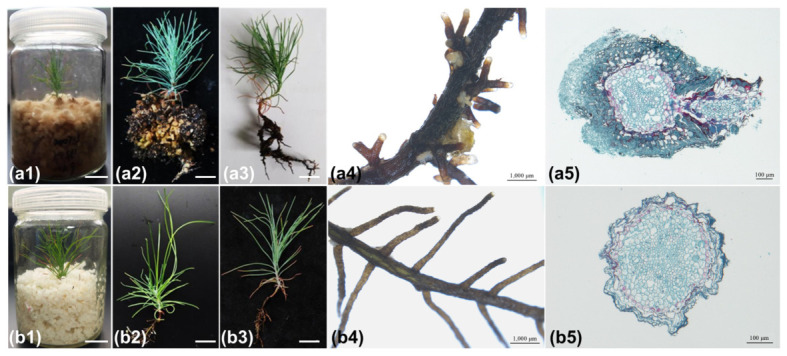
External morphology and anatomical structure of mycorrhiza formed by regenerated plants of *Pisolithus orientalis* cfcc7668 and *Pinus elliottii*. (**a1**) Culture vessel of an inoculated plantlet showing extensive fungal mycelium forming a dense network throughout the perlite-based substrate. (**a2**) Root system covered by mycelium-coated perlite particles. (**a3**,**a4**) Characteristic coralloid, dichotomously branched mycorrhizal roots after gentle removal of adhering perlite. (**a5**) Transverse section of an inoculated root showing a well-developed Hartig net along the cortical cell layer and surrounded by an outer fungal mantle, with the original cortical cells mostly preserved but appearing thin. (**b1**) Non-inoculated control plantlet in axenic perlite with no visible fungal growth. (**b2**–**b4**) Roots of non-inoculated plantlets, which remain thin, pale, and predominantly monopodial with limited lateral branching. (**b5**) Transverse section of a non-inoculated control root showing typical root anatomy, with thin cortical cells and no fungal structures. (**a1**–**a3**,**b1**–**b3**) Bar = 1 cm; (**a4**,**b4**) Bar = 1000 µm; (**a5**,**b5**) Bar = 100 µm.

Following in vitro inoculation with *P. orientalis* cfcc7668, extensive fungal colonization was observed in the culture vessels, with mycelium forming a dense network throughout the perlite-based substrate (Figure 3(a1)). In contrast, non-inoculated controls grown under axenic conditions showed no visible fungal growth (Figure 3(b1)). Upon removal from the culture vessels, inoculated plantlets exhibited root systems covered by mycelium-coated perlite particles (Figure 3(a2)). After gentle removal of adhering perlite particles, the roots displayed a grey-brown appearance and a characteristic coralloid, dichotomously branched morphology (Figure 3(a3,a4)), consistent with ectomycorrhizal formation. In contrast, roots of non-inoculated plantlets remained thin, pale, and predominantly monopodial, with limited lateral branching (Figure 3(b2–b4)). Transverse sections of inoculated roots confirmed the establishment of a functional ectomycorrhizal interface. A well-developed Hartig net was clearly visible, with fungal hyphae penetrating between cortical cells (Figure 3(a5)). Such structures were absent in roots of non-inoculated controls, which displayed typical root anatomy without fungal association (Figure 3(b5)). Quantification of the mycorrhizal association revealed a mycorrhization rate of 97.7 ± 1.1% and a mycorrhizal infection rate of 88.2 ± 1.2% at 90 days after inoculation.

**Figure 4:**
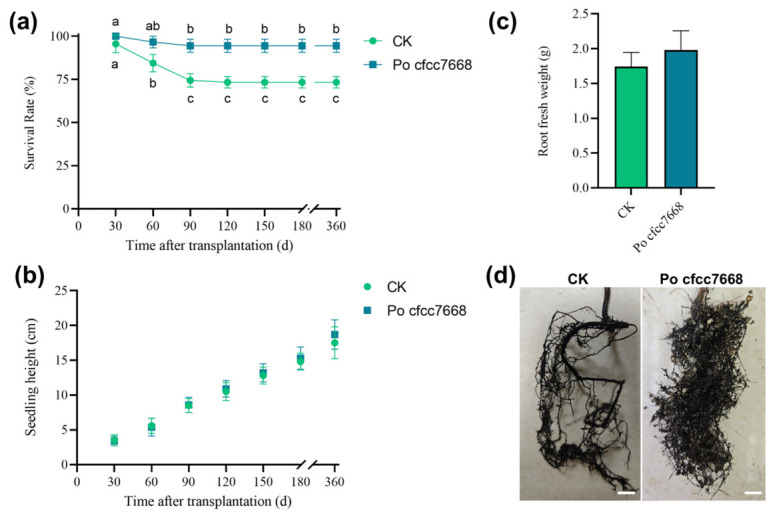
Effects of mycorrhization on *Pinus elliottii* plantlet transplantation. (**a**) Survival rates over 360 days. CK, non-inoculated control; Po cfcc7668, mycorrhizal treatment. (**b**) Seedling heights. (**c**) Fresh root weight at 360 days. (**d**) Representative root morphology of control and mycorrhizal plantlets 360 days after transplantation. Data are mean ± SD. Different letters in (**a**) indicate significant differences (*p* < 0.05, Šidák’s test). No significant differences were found in shoot height (**b**) or root fresh weight (**c**). Bar = 1.0 cm.

Following transplantation into the optimal 3:1:1 substrate, survival was monitored for 360 days. Non-inoculated control plantlets showed an initial survival rate of 95.6 ± 5.1% at 30 days, which declined to 84.4% by 60 days and stabilized at approximately 73.3% from 90 days onward. In contrast, mycorrhizal plantlets achieved 100% survival at 30 days and remained above 94% throughout the 360-day observation period (Figure 4a). Two-way ANOVA revealed that both treatment and time had significant effects on survival (treatment: F_1,28_ = 224.2, *p* < 0.0001; time: F_6,28_ = 12.41, *p* < 0.0001). The interaction between treatment and time was also significant (F_6,28_ = 4.57, *p* = 0.0024). Šidák’s multiple comparisons test indicated that survival did not differ between the two groups at 30 days (*p* = 0.7), but became significantly higher in mycorrhizal plantlets from 60 days onward (*p* = 0.003 at 60 days, *p* < 0.0001 for 90–360 days; Figure 4a).

Shoot height was recorded at the same time points. Both mycorrhizal and control plantlets increased steadily in height, reaching 18.7 ± 2.1 cm and 17.5 ± 2.3 cm, respectively, at 360 days, with no significant differences between the two groups at any time point (Figure 4b). Two-way ANOVA for shoot height showed no significant effect of treatment (F_1,28_ = 0.61, *p* = 0.44) and no treatment × time interaction (F_6,28_ = 0.18, *p* = 0.99), while time had a significant effect (F_6,28_ = 87.2, *p* < 0.0001). The fresh root weight of mycorrhizal roots (1.98 ± 0.28 g) was slightly higher than that of controls (1.74 ± 0.21 g), but the difference was not statistically significant (*p* = 0.16; Figure 4c). Representative root systems are shown in Figure 4d. These results demonstrate that mycorrhizal inoculation significantly improves long-term transplant survival after the first month, even though it does not affect shoot height or fresh weight under the present greenhouse conditions.

## 3. Discussion

Our study demonstrates that the low and variable survival rates of somatic embryo-derived conifers during acclimatization represent a tractable challenge that can be addressed through a systematic approach. By sequentially improving plantlet quality, environmental conditions, and ectomycorrhizal establishment, we achieved 100% survival at 30 days and sustained high survival over 360 days. This strategy directly addresses a key bottleneck that has limited the operational deployment of SE, a technology essential for advanced breeding and large-scale reforestation in pines [3]. This challenge is situated within a broader, ongoing scientific effort to understand and optimize the multi-step somatic embryogenesis process across the diversity of conifers [34]. Our integrated protocol therefore provides a potential practical solution to bridge the gap between laboratory potential and greenhouse application under the conditions tested.

The foundation of our protocol was the systematic production of high-quality plantlets. The superior performance of plantlets derived from our optimized maturation protocol is consistent with the established role of ABA in promoting storage reserve accumulation and stress-related proteins, which has been specifically optimized to enhance embryo quality in *Pinus* species [35,36]. This internal “pre-hardening” is crucial, as the physiological state of the host plant significantly influences its capacity to form a successful symbiosis [37]. The beneficial effect of glucose we observed during germination may reflect its direct role in energy metabolism and more rapid energy mobilization [38]. Selecting the most vigorous plantlets further ensured that only the most competent individuals advanced to the challenging transplantation stage.

While high-quality plantlets are essential, they remain vulnerable to the harsh ex vitro environment. Our finding that a peat-rich substrate (3:1:1 ratio) was optimal highlights the critical importance of the physical environment as the first line of defense. Such a substrate provides an ideal balance between water-holding capacity and aeration, mitigating initial water stress and facilitating root exploration [25]. This optimization pushed the survival rate of control plantlets to 98.9 ± 1.9% (Table 1), representing the practical ceiling achievable through conventional environmental means alone.

A key finding of this study is that the transition to 100% survival was achieved not through further refinement of the physical environment, but through the introduction of pre-established symbiosis with *P. orientalis* cfcc7668. This reflects a conceptual shift from passive protection of the plantlet to active biological support. The anatomical transformation of the root system from a sparse, monopodial structure to a densely branched, coralloid morphology is a well-established indicator of ectomycorrhizal formation and a substantial increase in functional capacity [28,39]. This extensive external mycelium acts as a surrogate root system, effectively increasing the surface area for water [40] and nutrient absorption [41,42].

The positive effect of ectomycorrhizal inoculation on transplant survival is well documented in the literature. For *P. pinea*, Ragonezi et al. showed that *P. arhizus* increased the survival rate of regenerated plantlets [28]. Similarly, Oliveira et al. obtained comparable results for *P. pinea* [27]. More recently, Chen et al. demonstrated a marked increase from 37% to 85% survival in mycorrhizal *P. massoniana* plantlets [31]. Our study extends these findings by showing that *P. orientalis* cfcc7668 not only raises survival to 100% in the short term but also sustains it above 94% for one year.

Beyond this physical extension, the fungal symbiont also functions as a biochemical partner. Ectomycorrhizal fungi are known to produce plant growth regulators like auxins and cytokinins, which can directly stimulate root cell division and branching [43]. This symbiotic interaction is initiated by the fungal partner, which undergoes early transcriptional reprogramming during symbiosis establishment [44]. Furthermore, ectomycorrhizal fungi confer bioprotection by exuding antimicrobial compounds and fostering a beneficial microbial community in the mycorrhizosphere, thereby reducing pathogen pressure on young roots [45]. The presence of a well-developed Hartig net provides anatomical evidence for the functional interface mediating resource and signal exchange between the symbiotic partners [46]. Consequently, the inoculated plantlets entered the greenhouse not as sterile individuals, but as holobionts with an established symbiotic status. This provides a mechanistic explanation for their complete survival and enhanced vigor.

Our findings must also be considered in the context of the broader challenges facing pine SE. Somatic seedlings are widely reported to exhibit inferior initial growth and vigor compared to their zygotic counterparts, representing a major limitation for breeding programs [47,48]. This “initial growth lag” has been observed in field trials of several commercially important pines, including *P. pinaster*, *P. taeda*, and *P. radiata*. It has been linked to suboptimal biological and physiological characteristics of the cotyledonary somatic embryos produced under current maturation protocols [3,47,49]. While some studies indicate that somatic clones can recover from this initial lag after several years in the field [47], a suboptimal start is likely to delay the realization of full genetic gains. Our bio-enhancement strategy, which targets the development of a functional root system immediately upon transplantation, offers a possible practical approach to mitigate this problem in slash pine. By ensuring near-complete survival and promoting early establishment vigor under the tested conditions, this approach improves the initial performance of somatic seedlings of this genotype.

This bio-enhancement strategy could have important implications for conifer biotechnology if confirmed in more other genotypes. It provides a potentially reproducible framework for addressing the acclimatization bottleneck in slash pine that has limited the practical application of many otherwise successful regeneration systems [15,28]. Future work should further investigate the molecular interactions during the in vitro co-culture phase and examine the specificity between host genotypes and fungal strains [32,50]. Screening a broader array of ectomycorrhizal fungi may enable more effective matching of symbionts to specific tree clones and target environments, thereby facilitating the operational deployment of somatic embryogenesis in sustainable forestry.

Our results were obtained with a single embryogenic line (2007-2); further studies are needed to test the protocol’s effectiveness across multiple genotypes and under field conditions.

## 4. Materials and Methods

### 4.1. Plant Material and Production of High-Quality Somatic Plantlets

Somatic embryo-derived plantlets of brown spot needle blight-resistant *P. elliottii* were used. The embryogenic cell line 2007-2 was subjected to an optimal maturation protocol based on previous work [10]. Specifically, to enhance embryo maturation competence, embryogenic suspension cultures were pre-treated with 5 mg/L abscisic acid (ABA) and 5 g/L maltose for three sequential one-week cycles before being plated onto solid maturation medium. The solid maturation medium consisted of LP medium (Lepoivre medium) [51] supplemented with 2 mg/L ABA, 1.5 mg/L GA3, 130 g/L PEG 8000, 3% (*w*/*v*) maltose, 1 g/L myo-inositol, 500 mg/L casein hydrolysate, 250 mg/L MES, and 500 mg/L L-glutamine. This approach (three ABA pre-treatments) has been demonstrated to significantly improve somatic embryo yield in *P. elliottii* [10]. After 75 days of culture on solid maturation medium, cotyledonary somatic embryos were obtained. For embryo maturation, 90 mm Petri dishes were used, each inoculated with 3 mL of settled embryogenic suspension. Mature somatic embryos were then germinated on DCR medium (Douglas–fir cotyledon revised medium) [52] with 20 g/L glucose (filter-sterilized) without plant growth regulators (PGRs). One month later, somatic embryos were transferred to rooting medium DCR basal with 0.1 mg/L NAA and 1.0 mg/L IBA. Finally, robust plantlets of 2.5~4.0 cm in height were selected for all subsequent acclimatization and bio-enhancement experiments.

All cultures were kept at 23 ± 2 °C. Embryo maturation and germination were conducted in darkness, whereas rooting and plantlet growth were under a 16 h light/8 h dark photoperiod (cool white fluorescent light, 40 μmol·m^−2^·s^−1^).

### 4.2. Transplant Substrate Optimization

Prior to transplantation, the caps of the culture vessels containing the regenerated plantlets were gradually loosened over one week to acclimatize the plantlets to ex vitro conditions. Uniform plantlets from cell line 2007-2 were transplanted into plastic pots (7.6 cm in diameter, 9 cm in height) containing three different substrates with varying ratios of peat, vermiculite, and perlite (1:1:1, 2:1:1, and 3:1:1, *v*/*v*/*v*). The experiment was set up in a completely randomized design with three independent replicates (biological repeats) per substrate treatment. Each replicate consisted of 30 plantlets, resulting in a total of 90 plantlets per substrate type. Pots were randomly arranged on greenhouse shelves. The peat:vermiculite:perlite mixture was used without autoclaving. After transplantation, all plantlets were placed on shelves in a growth room under a 16 h photoperiod provided by cool white fluorescent tubes (120 μmol·m^−2^·s^−1^), a constant temperature of 21 ± 2 °C, and relative humidity of 70–80%. No fertilizers were applied. Each pot was watered once per week with tap water. Survival was assessed after 30 days.

### 4.3. Ectomycorrhizal Inoculation and Acclimatization

For the mycorrhization experiment, uniform plantlets from cell line 2007-2 were first transferred to a perlite substrate supplemented with DCR nutrient solution. They were then randomly divided into two groups per experimental run: 30 plantlets served as the non-inoculated control, and the other 30 plantlets were each inoculated with three mycelial plugs of *P. orientalis* cfcc7668. The fungal strain cfcc7668 was obtained from the China Forestry Culture Collection Center (CFCC), originally supplied as *Pisolithus tinctorius*. To confirm its identity, we performed ITS sequencing and phylogenetic analysis. The ITS sequence of cfcc7668 was aligned with reference sequences of *Pisolithus* species. In the resulting phylogenetic tree), cfcc7668 clustered with *P. orientalis* reference strains, including the holotype, and was clearly separated from the *P. tinctorius* clade. We therefore refer to the strain as *P. orientalis* cfcc7668 in this paper. The perlite co-culture system was checked weekly for contamination. Any vessel showing microbial growth would be discarded. The experiment was performed in three independent runs, resulting in a total of 90 plantlets per treatment (180 plantlets in total). After 90 days of co-culture, the plantlets were acclimatized for one week by gradually loosening the caps of the culture vessels. They were then transplanted into the optimal 3:1:1 substrate in plastic pots (same size as in Section 4.2). After transplantation, the plantlets were maintained under the same growth room conditions and watering regime (once per week with tap water, no fertilizers) as described in Section 4.2. Survival rates and shoot height were recorded at 30, 60, 90, 120, 150, 180 and 360 days after transplantation. At 360 days, root fresh weight was measured from five randomly selected plantlets per treatment. Mycorrhization rate was calculated as (number of colonized plants/total number of inoculated plants) × 100%. The mycorrhizal infection rate was determined by examining at least 20 root segments per plantlet; a root segment was considered colonized when a Hartig net or mantle was observed. The rate was calculated as (colonized root segments/total segments) × 100%. External root morphology was documented using a Zeiss SteREO Discovery V20 ste-reomicroscope (Carl Zeiss Microscopy GmbH, Oberkochen, Germany).

### 4.4. Anatomical Observation of Mycorrhizal Structures

Root samples were collected from at least five randomly selected inoculated and non-inoculated plantlets per experimental run at 90 days after inoculation. Samples were fixed in FAA solution, dehydrated through a graded ethanol series, embedded in paraffin, sectioned, and stained with safranin and fast green. Sections were examined under a Zeiss Axio Imager M2 light microscope (Carl Zeiss Microscopy GmbH, Göttingen, Germany) to assess the formation of the Hartig net.

### 4.5. Data Collection and Statistical Analysis

The experiment was conducted using a completely randomized design. No data were missing; all plantlets that survived were included in the analysis. Biological replicates were three independent runs (each with 30 plantlets per treatment). Data on survival rate, plant height, and root fresh weight were collected. For substrate optimization, one-way analysis of variance (ANOVA) was performed, followed by Duncan’s multiple range test (*p* ≤ 0.05). For survival rate and shoot height measured repeatedly over time (30–360 days), two-way ANOVA with treatment and time as factors was applied, followed by Šidák’s multiple comparisons test to compare the two treatments at each time point. For root fresh weight at 360 days (two groups), Student’s *t*-test was used. All data were analyzed using SPSS Statistics 17.0 (IBM Corp., Armonk, NY, USA) and GraphPad Prism 9.5.0 (GraphPad Software, Boston, MA, USA). A significance level of *p* ≤ 0.05 was considered statistically significant.

## 5. Conclusions

We improved survival of somatic embryo-derived *P. elliottii* plantlets by optimizing plantlet production and by inoculating with ectomycorrhizal fungi before transplanting. The optimized maturation and germination steps produced vigorous plantlets. Inoculation with *P. orientalis* cfcc7668 gave 97.7% mycorrhization, raised transplant survival to 100% at 30 days, and maintained above 94% over 360 days, whereas non-inoculated controls declined to about 73%. Mechanistically, the mycorrhizal symbiosis enhances root water and nutrient uptake through the extraradical mycelium and the Hartig net, while also offering bioprotection against soil pathogens. Under the specific conditions tested (single clone, small pots, no fertilization), inoculating plantlets with ectomycorrhizal fungi before transplantation is a practical way to overcome a major bottleneck in conifer somatic embryogenesis and to produce healthy planting stock. Its generalizability to other genotypes and field conditions warrants further investigation.

## Figures and Tables

**Table 1:** Effects of different substrate compositions on the growth of regenerated *P. elliottii* plantlets.

Substrate (Peat:Vermiculite:Perlite)	Survival Rate (%)
1:1:1	66.7 ± 12.0 b
2:1:1	93.3 ± 3.3 a
3:1:1	98.9 ± 1.9 a

Notes: Means followed by different letters within a column are significantly different (*p* ≤ 0.05). Values are mean ± SD of three independent replicates (30 plantlets per replicate, total 90 plantlets per substrate type).

## Data Availability

All data generated or analyzed during this study are included in this article.
